# Smith-Waterman peak alignment for comprehensive two-dimensional gas chromatography-mass spectrometry

**DOI:** 10.1186/1471-2105-12-235

**Published:** 2011-06-15

**Authors:** Seongho Kim, Imhoi Koo, Aiqin Fang, Xiang Zhang

**Affiliations:** 1 Department of Bioinformatics and Biostatistics, University of Louisville, Louisville, KY 40292, USA; 2 Department of Chemistry, University of Louisville, Louisville, KY 40292, USA

## Abstract

**Background:**

Comprehensive two-dimensional gas chromatography coupled with mass spectrometry (GC × GC-MS) is a powerful technique which has gained increasing attention over the last two decades. The GC × GC-MS provides much increased separation capacity, chemical selectivity and sensitivity for complex sample analysis and brings more accurate information about compound retention times and mass spectra. Despite these advantages, the retention times of the resolved peaks on the two-dimensional gas chromatographic columns are always shifted due to experimental variations, introducing difficulty in the data processing for metabolomics analysis. Therefore, the retention time variation must be adjusted in order to compare multiple metabolic profiles obtained from different conditions.

**Results:**

We developed novel peak alignment algorithms for both homogeneous (acquired under the identical experimental conditions) and heterogeneous (acquired under the different experimental conditions) GC × GC-MS data using modified Smith-Waterman local alignment algorithms along with mass spectral similarity. Compared with literature reported algorithms, the proposed algorithms eliminated the detection of landmark peaks and the usage of retention time transformation. Furthermore, an automated peak alignment software package was established by implementing a likelihood function for optimal peak alignment.

**Conclusions:**

The proposed Smith-Waterman local alignment-based algorithms are capable of aligning both the homogeneous and heterogeneous data of multiple GC × GC-MS experiments without the transformation of retention times and the selection of landmark peaks. An optimal version of the SW-based algorithms was also established based on the associated likelihood function for the automatic peak alignment. The proposed alignment algorithms outperform the literature reported alignment method by analyzing the experiment data of a mixture of compound standards and a metabolite extract of mouse plasma with spiked-in compound standards.

## Background

Metabolomics examines the structures, functions, interactions, and dynamics of cellular metabolites, identifies their cellular localization (i.e., subcellular membrane compartments and domains), and determines the dynamic changes that occur during physiological and pathophysiological perturbations. Metabolomics, together with other modern omics, has the potential to facilitate the development of preventive, predictive and personalized medicine markets in health and wellness [1]. Multiple high-throughput analytical methods have been developed for metabolomics. One such powerful approach is comprehensive two-dimensional gas chromatography-mass spectrometry (GC × GC-MS) [2-5], which can easily detect a large number of metabolites from a complex sample.

The GC × GC-MS system provides much increased separation capacity, chemical selectivity and sensitivity for metabolomics analyses [6-13]. This approach uses two columns connected via a thermal modulator. Its second column is usually a short column after the main analytical column, where the second column is naturally operated at a higher temperature than the first column with different stationary phase. The compounds co-eluted from the first column are further separated in the second column through the difference of column temperature and the chromatographic matrix. The further separated compounds are directed to a high capacity time-of-flight mass spectrometry system for detection. Consequently, the GC × GC-MS system brings more accurate information about compound retention times and mass spectrum, representing a powerful technique for the analysis of compounds in complex biological systems. However, as in one-dimensional GC, retention times are shifted due to uncontrollable factors such as temperature and pressure fluctuations, matrix effects on samples and stationary phase degradation. As a result, it is difficult to compare compound profiles obtained from multiple samples.

To date, six alignment methods have been published to correct the retention time shifts in the GC × GC system. For aligning data in small or local regions, Fraga et al. [14] proposed a generalized rank annihilation method and Mispelaar et al. [15] introduced a correlation-optimized shifting method. The alignment algorithm for the entire chromatogram of GC × GC-MS data was developed by Pierce et al. [16] using an indexing scheme with a piecewise retention time alignment algorithm. Zhang et al. [17] developed a two-dimensional correlation optimized warping method (2-D COW). These four methods align the GC × GC-MS data only based on two-dimensional retention times without using the signature feature of a compound, i.e., mass spectrum of fragment ions. Therefore, it is likely that a high rate of false-positive alignment is introduced due to the fact that some compounds with similar chemical functional groups have similar retention times in the two gas chromatographic dimensions.

Oh et al. [18] and Wang et al. [19] developed peak-based alignment methods using the two-dimensional retention times as well as the mass spectrum of compound fragment ions, which are called MSort and DISCO, respectively. In these methods, the raw instrument data of each sample were first reduced to a compound peak list, where each compound was characterized by its two-dimensional retention times, mass spectrum and other features. The two-dimensional retention times and mass spectra were then used for compound alignment. Incorporating compound mass spectrum into alignment can greatly reduce the rate of false-positive alignment. DISCO can align both homogeneous and heterogeneous data while MSort can be applied only to homogeneous data. The homogeneous data refer to experiment data acquired under the identical GC × GC-MS experiment conditions and the heterogeneous data acquired under different GC × GC-MS conditions.

In order to enable the analysis of heterogeneous data, DISCO first transfers the two-dimensional retention time values to z-scores, to balance the contribution of the two-dimensional retention times to the Euclidean distance between two peaks. It then selects a number of landmark peaks and corrects the retention times of the rest of peaks based on these selected landmark peaks. The landmark peaks are peaks that appear in all samples (peak lists), and are discovered by an optimization process followed by a filtering process. The optimization process employs the Euclidean distance of two peaks in the two-dimensional retention time space and fragment ion mass spectra were employed in sequential, while the filter process removes false landmark peaks based on compound elution order in the first and the second dimension GC, respectively. After landmark peak discovery, a local linear fitting is rendered to the remaining peaks based on the selected landmark peaks. However, it is more likely that the retention time shift is nonlinear in reality [20]. The performance of DISCO algorithms highly depends on the accuracy of landmark peak selection and the local linear fitting approach may not able to precisely adjust compound retention time due to the nature of nonlinear retention time shift. For this reason, we developed novel peak alignment algorithms to align homogeneous as well as heterogeneous data using Smith-Waterman local alignment, in which the landmark peak selection and retention time transformation are not required. All the statistical analyses and simulations were performed using a statistical package R (R Development Core Team) and the R code is available at http://stage.louisville.edu/faculty/x0zhan17/home.html.

## Method

### GC × GC-MS data

In this study, two sets of GC × GC-MS data were used. One is a mixture of 116 compound standards and the other is a metabolite extract with spiked-in compounds. In the first dataset (Dataset I), a mixture of 76 compound standards (8270 MegaMix, Restek Corp., Bellefonte, PA), C7-C40 saturated alkanes (Sigma-Aldrich Corp., St. Louis, MO) and a deuterated six component semi-volatiles internal standard (ISTDF) mixture (Restek Corp., Bellefonte, PA) at a concentration of 2.5 μg/mL were analyzed on a LECO Pegasus 4D GC × GC-MS instrument (LECO Corporation, St. Joseph, MI, USA) equipped with a cryogenic modulator. The GC × GC-MS analyses were repeated 10, 2, and 4 times under three different temperatures, 5°C/min, 7°C/min, and 10°C/min, respectively, resulting in a total of 16 datasets.

As for the spiked-in sample (Dataset II), a 100 μL mouse plasma sample was mixed with 900 μL of organic solvent mixture (methanol/water 8:1, v/v) and vortexed for 15 s. After sitting at 20°C for 30 min, the mixture was centrifuged with 16000 ×g at 4°C for 15 min. Supernatants from the mixture were collected and evaporated to dryness with a SpeedVac and then redissolved in 100 μL of pyridine. Fifty micro litters of the metabolite extract were treated with 100 μL of 50 mg/mL ethoxyamine hydrochloride pyridine solution for 30 min at 60°C. Subsequently, the extracts were derivatized with 100 μL of MTBSTFA for 1 h at 60°C. The derivatized sample was spiked with ISTD mixture at a concentration of 2.5 μg/mL right before the GC × GC-MS analysis. Then the compounds were analyzed five times on GC × GC-MS.

All GC × GC/TOF-MS analyses were performed on a LECO Pegasus 4D time-of-flight mass spectrometer (TOF-MS) with a Gerstel MPS2 autosampler. The Pegasus 4D GC × GC/TOF-MS instrument was equipped with an Agilent 7890 gas chromatograph featuring a LECO two-stage cryogenic modulator and secondary oven. A 30 m × 0.25 mm i.d. × 0.25 μm film thickness, Rxi-5 ms GC capillary column (Restek Corp., Bellefonte, PA) was used as the primary column for the GC × GC/TOF-MS analysis. A second GC column of 1.2 m × 0.10 mm i.d. × 0.10 μm film thickness, BPX-50 (SGE Incorporated, Austin, TX) was placed inside the secondary GC oven after the thermal modulator. The helium carrier gas flow rate was set to 1.0 mL/min at a corrected constant flow via pressure ramps. A 1 μL liquid sample was injected into the linear using the splitless mode with the injection port temperature set at 260°C. The primary column temperature was programmed with an initial temperature of 60°C for 0.5 min and then ramped at a variable temperature gradient to 315°C. The secondary column temperature program was set to an initial temperature of 65°C for 0.5 min and then also ramped at the same temperature gradient employed in the first column to 320°C accordingly. The thermal modulator was set to +20°C relative to the primary oven, and a modulation time of 5 s was used. The MS mass range was 10-750 *m*/*z *with an acquisition rate of 150 spectra per second. The ion source chamber was set at 230°C with the MS transfer line temperature set to 260°C, and the detector voltage was 1800 V with electron energy of 70 eV.

The LECO ChromaTOF software version 3.4 equipped with the National Institute of Standards and Technology MS database (NIST MS Search 2.0, NIST/EPA/NIH Mass Spectral Library, NIST 2002) was used for instrument control, spectrum deconvolution, and compound identification. Manufacturer recommended parameters for ChromaTOF were used to reduce the raw instrument data into a compound peak list. These parameters are: baseline offset = 0.5; smoothing = auto; peak width in first dimension = 6 s; peak width in the second dimension = 0.1 s; signal-to-noise ratio = 100; match required to combine peaks = 500; R.T. shift = 0.08 s; minimum forward similarity match = 600. The peak list of each GC × GC-MS data was then manually examined. In case that there are multiple peaks identified as the same compound in an experiment, only the peak with the largest peak areas was selected. Table 1 summarizes each dataset by calculating the number of compounds. The numbers in parentheses are the original number of peaks before correcting the multiple peaks. The scatter plots of Dataset I and II, the density plots of the first and second dimension retention times are depicted in Figure S1 as given in the Additional File 1. Since the identified compounds by ChromaTOF could be wrong, all the compound names identified are "tentative."

**Table 1:** The summary of GC × GC/TOF-MS datasets.

(a) Compound standards 5°C/min											
	
RUN ID	S1	S2	S3	S4	S5	S6	S7	S8	S9	S10	
	
The number of compounds	78 (180)*	76 (186)	76 (161)	75 (151)	74 (151)	73 (145)	74 (172)	76 (163)	77 (168)	75 (174)	
											

	**Compound standards** **(continued)**										
	**7°C/min**		**10°C/min**				**(b) Spiked-in**				
			
**RUN ID**	**S11**	**S12**	**S13**	**S14**	**S15**	**S16**	**C1**	**C2**	**C3**	**C4**	**C5**
			
**The number of compounds**	75 (132)	73 (170)	76 (148)	73 (138)	76 (113)	75 (118)	466 (759)	456 (733)	436 (694)	452 (727)	418 (661)

*, the number of peaks found by ChromaTOF before multiple peak correction

(a) A total of 16 datasets were generated under the temperature gradients of 5°C/min, 7°C/min, and 10°C/min for the mixture of compound standards. (b) A total of 5 datasets were generated for spiked-in sample.

### Similarity measure

The most widely used mass spectral similarity measures are the Finnigan INCOS dot product and the probability based matching (PBM) [21,22]. Stein and Scott (1994) demonstrated that the dot product is the best performed measure out of five similarity measures including PBM. On the other hand, Liu et al. [23] compared different measures of spectral similarity and concluded that the Pearson's correlation coefficient is robust but the difference between the dot product and the Pearson's correlation coefficient is subtle. In this study, we used the Pearson's correlation coefficient for the purpose of comparison with DISCO algorithms, in which the Pearson's correlation coefficient was employed.

The Pearson's correlation coefficient for mass spectral similarity measure between two mass spectra,  and , of two peaks, *y_j _*and *x_i_*, as follows:

### Smith-Waterman local alignment

The Smith-Waterman (SW) local alignment was introduced by Temple Smith and Michael Waterman for the identification of common molecular subsequences, where the optimal local alignment between two sequences was determined by calculating the similarity score using dynamic programming [24,25]. The SW algorithm is closely related to global alignment, i.e., Needleman-Wunsch global alignment [26].

Consider two sequences *X *= *x*_1 _*x*_2 _⋯ *x_m _*of length *m *and of *Y *= *y*_1_*y*_2 _⋯ *y_n _*of length *n*. For 1 ≤ *h *≤ *i *≤ *m *and 1 ≤ *k *≤ *j *≤ *n*, we denote by *X_h_*, *_j _*and *Y_k_*, *_j _*the sub-sequences of *X *and *Y *given by *x_h_x_h_*_+1 _⋯ *x_i _*and *y_k_y_k_*_+1 _⋯ *y_j_*, respectively, and by *H*(*i, j*) the maximum of all possible scores for alignments between a sub-sequence of *X *ending at *x_i _*and one of *Y *ending at *y_j_*. In particular, *H*(*i, j*) is set to zero when *H*(*i, j*) is negative. The SW algorithm uses dynamic programming, by initializing

and by calculating(1)

to find the maximum *H*(*i, j*) of over all values of *i *and *j*, where *m*(*i, j*) = *u *if *x_i _*= *y_j _*and *v *otherwise and *d *is the gap penalty for some non-negative constants *u*, *v*, *d*.

To find the highest-scoring alignment, the path of choices from (1) should be found using the procedure called a *traceback*. The traceback procedure works by building the alignment in reverse, i.e., starting from the highest value of *H*(*i, j*)and ending at a cell with a value of zero. The overview and variants of the SW algorithm have been described in great detail by Ewens and Grant [25].

### Pairwise peak alignment implementation

All the pairs of peak lists among the datasets were constructed, considering that one was a reference chromatogram and the other was a target chromatogram. For the comparison analysis of homogeneous peak alignment, a total of 45 homogeneous chromatogram pairs were generated by compound standards with a temperature gradient of 5°C/min and 10 homogeneous chromatogram pairs by the spiked-in sample. As for heterogeneous peak alignment, the pairs were created between (5°C/min and 7°C/min), (5°C/min and 10°C/min), and (7°C/min and 10°C/min) using compound standards data, resulting in a total of 68 heterogeneous chromatogram pairs.

### The comparison criterion

The performances of all the methods are compared by calculating the true positive rate (TPR), positive predictive value (PPV), and F1 score of the peak alignment.

Suppose there are *n *target peaks *Y *= {*y*_1_, *y*_2_, ... *y_r_*, *y_r_*_+1_, ..., *y_n_*} and *m *reference peaks *X *= {*x*_1_, *x*_2_, ..., *x_r_*, *x_r_*_+1_, ..., *x_m_*} with *r *positive peak pairs {(*y*_1_, *x*_1_), (*y*_2_, *x*_2_), ..., (*y*_r_, *x*_r_)}, where *r *≤ *min*(*n*, *m*). Note that if two peaks are generated by the same compound, it is called a positive peak pair. If a certain peak alignment method is used for the two datasets, Y and X, to find *t *peak pairs matched, then the values of TPR and PPV of the peak alignment between two datasets are calculated by the following equations:

where TP is the number of positive peak pairs that were aligned as positive (true positive) and is less than or equal to *min*(*r*, *t*), FP is the number of negative peak pairs that were aligned as positive (false positive) and is *t *- TP, FN is the number of positive peak pairs that were not aligned (false negative) and is *r *- *TP*, and TN is the number of negative peaks that were not aligned (true negative) and is *m*·*n *- *r *- FP. Note that the total number of peak pairs is *m*·*n*.

TPR is called recall and PPV precision. Their harmonic mean  is then used as an accuracy which is called F1 score. F1 score was used as the accuracy measure of the peak alignment. Thus, the larger are TPR and PPV, the larger is F1 score. That is, if F1 score (or TPR and PPV) is larger, the peak alignment performs better.

## Results

### Smith-Waterman peak alignment algorithms

The Smith-Waterman (SW) local alignment was originally developed for the alignment of gene sequences [20]. We present modified SW algorithms that support the peak alignment based on the peak list of comprehensive two-dimensional gas chromatography mass spectrometry data. The details of SW algorithm are described in the Method section.

We use the following notations throughout the article. Let *Y *= *y*_1_*y*_2 _⋯ *y_n _*be the ordered peak list of the target GC × GC-MS data and *X *= *x*_1_*x*_2 _⋯ *x_m _*the ordered peak list of the reference GC × GC-MS, where *x_i _*and *y_j _*(1 ≤ *i *≤ *m*, 1 ≤ *j *≤ *n*) are composed of the first and the second retention times of the *i*th and *j*th peaks, (*x_i_*_,1, _*x_i_*_,2_) and (*y_j_*_,1, _*y_j_*_,2_), respectively. That is, both *X *and *Y *are sorted in ascending order of the sum of two retention times, *x_i_*_,1 _+ *x_i_*_,2 _and *y_j_*_,1 _+ *y_j_*_,2_, for 1 ≤ *i *≤ *m *and 1 ≤ *j *≤ *n*, respectively. We denote by *Y_k, j _*and *X_h, i _*the sublists of the ordered peak lists, *Y *and *X*, of the target and reference GC × GC-MS data given by *y_k_y_k_*_+1 _⋯ *y_j _*and *x_h_x_h_*_+1 _⋯ *x_i _*for 1 ≤ *k *≤ *j *≤ *n*, and 1 ≤ *h *≤ *i *≤ *m*, respectively.

A similarity *w*(*i, j*) function is defined as follows:(2)

where *u *and *v *are non-negative constants, and *ρ *is a user-defined cut-off value for the mass spectral similarity ranging between 0 and 1. Note that we employed the Pearson's correlation coefficient for *S*(*x_i_, y_j_*) as described in the Method section. Then, by replacing *m*(*i, j*) in (1) with *w*(*i, j*) in (2), the peak alignment can be rendered using the SW algorithm. Since its traceback will be stopped when encountering a zero, the SW algorithm will give the single local match between two peak lists. For this reason, we propose three modified SW algorithms by changing its traceback process to find all the possible local peak alignments with a significant score.

Once the *m *× *n *score matrix is constructed using (2), the traceback is rendered after finding the maximum value of *H*(*i, j*)over all values of *i *and *j*, where 1 ≤ *i *≤ *m *and 1 ≤ *j *≤ *n*. Let us assume that the highest value occurs at the cell (*q, r*). Then, at each step in the traceback process of 1 ≤ *i *≤ *q *≤ *m *and 1 ≤ *j *≤ *r *≤ *n*, the current cell (*i, j*) is moved back to the one of the cells (*i*-1, *j*-1), (*i*-1, *j*) or (*i*, *j*-1) by starting from which the highest value of *H*(*q, r*) was derived. At the same time, a pair of symbols is added onto the front of the current peak alignment: *x_i _*and *y_j _*if the step was to (*i*-1, *j*-1), *x_i _*and the gap character '-' if the step was to (*i*-1, *j*), or the gap character '-' and *y_j _*if the step was to (*i*, *j*-1). Then the traceback is ended when meeting a cell with the value of zero. The original traceback of the SW algorithm is stopped in this cell and outputs the best single local alignment. However, our modified traceback will find the path further until it reaches the start of the matrix, where *i *= 1 or *j *= 1. To do this, when the current traceback meets a cell (*s, t*) with the value of zero and the position of this cell is not the start of the score matrix, i.e., *s *≠ 1 and *t *≠ 1, the proposed algorithm finds the maximum value of *H*(*i, j*) over all values *i *and *j*, where 1 ≤ *i *≤ *s *≤ *q *≤ *m *and 1 ≤ *j *≤ *t *≤ *r *≤ *n*. If the cell (*v, w*) has the maximum value of *H*(*i, j*), the previous traceback is rendered similarly for 1 ≤ *i *≤ *s *and 1 ≤ *j *≤ *t *until meeting a cell with the value of zero or until it reaches the start of the matrix. If the current cell is the start of the matrix, the traceback process is stopped and, if not, the traceback will be rendered again. We call this modified SW algorithm the SW repeat alignment with maximum scores (SWRM).

In the second scheme, the traceback is first rendered from the last cell (*m, n*), while the traceback starts from the maximum value of *H*(*i, j*) in SWRM. If the cell (*s, t*) with the value of zero is not the start of the matrix, the traceback starts again from the cell (*s, t*) to find the path over all values of *i *and *j*, where 1 ≤ *i *≤ *s *and 1 ≤ *j *≤ *t*, and so on. We call this scheme the SW repeat alignment with ending scores (SWRE).

The maximum value of *H*(*i, j*) is estimated at the last column of the peak list of the target GC × GC-MS data. That is, we first look for the highest value of *H*(*i, n*) over all values of *i *and *j*, where 1 ≤ *i *≤ *m*. If the highest value is equal to zero, the maximum value of *H*(*i, j*) is found over all values of *i *and *j *for 1 ≤ *i *≤ *m *and *j *= *n *- 1. This process is repeated until the non-zero maximum value is found. Then the traceback is rendered from which the non-zero highest value was derived. We call this method the SW repeat alignment with maximum of ending scores (SWRME).

For each peak pair, the three proposed alignment algorithms and DISCO were implemented along with the different cut-off values of mass spectral similarity. Once a pair of the peak lists is aligned by the proposed algorithms, the peak pairs with mass spectral similarity greater than the cut-off value *ρ *are retained and the rest of peak pairs are discarded. The means and standard errors (SEs) of TPR, PPV, and F1 score for all the cases of each peak alignment method are estimated for the purpose of performance comparison. The results of this estimation are given in the Additional File 2.

### Homogeneous GC × GC-MS pairwise peak alignment

The proposed algorithms and DISCO algorithms were implemented for homogeneous GC × GC-MS data to examine their performance of the peak alignment. In this case, the two homogeneous GC × GC-MS data were utilized for the comparison analysis. One is the mixture of compound standards composed of 10 datasets of 5°C/min (its run id is from S1 to S10 as shown in Table 1) and the other is the spiked-in metabolite sample extracted from rat plasma, which is composed of 5 datasets (its run id is from C1 to C5 as shown in Table 1). The performance was compared based on the true positive rate (TPR), the positive predictive value (PPV), and the F1 score as described in the Method section. Since the performance of all the peak alignment algorithms here depends on the cut-off values determined by users, a total of 13 values were used for *ρ*, which are 0.1, 0.2, 0.3, 0.4, 0.5, 0.6, 0.7, 0.8, 0.9, 0.93, 0.95, 0.97, and 0.99, to examine its effect on the peak alignment.

**Figure 1:**
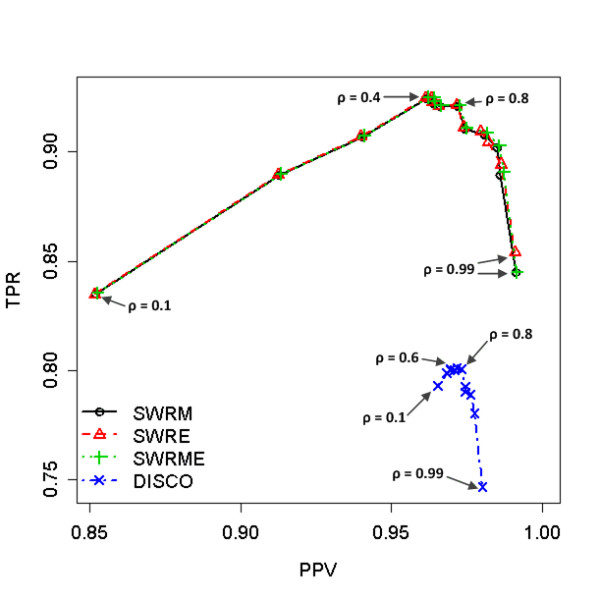
**The effect of cut-off values, ρ, in case of homogeneous data (Compound standards data)**. The true positive rates (TPR) versus the predictive positive values (PPV) are plotted. The performances of four different methods, SWRM, SWRE, SWRME, and DISCO, are compared according to the thirteen different values, (0.1, 0.2, 0.3, 0.4, 0.5, 0.6, 0.7, 0.8, 0.9, 0.93, 0.95, 0.97, 0.99). The maximum F1 scores are occurred at ρ = 0.8 for all the methods.

Figure 1 shows the performance of the peak alignment for the first data set based on TPR and PPV. It should be noted that the larger are TPR and PPV, the larger is Fl score. Thus, as Fl score (or TPR and PPV) is larger, the performance of peak alignment becomes better. All the methods, SWRM, SWRE, SWRME, and DISCO, have their maximum Fl score when *ρ *= 0.8 (as also shown in Figure S2 (a) in the Additional File 1). Of these four peak alignment algorithms, the SWRME method has the highest Fl score, but it is not significantly different from these of SWRM and SWRE when *ρ *= 0.8 (Fl score: SWRM = 0.9455 ± 0.0047; SWRE = 0.9457 ± 0.0048; SWRME = 0.9461 ± 0.0047). Interestingly, the Fl score of DISCO is significantly less than these of the proposed methods (Fl score = 0.8777 ± 0.0055). The maximum TPR is occurred when *ρ *= 0.4 in case of the proposed methods and, when *ρ *= 0.6, DISCO has the highest TPR (TPR: SWRM = 0.9245 ± 0.0059; SWRE = 0.9244 ± 0.0059; SWRME = 0.9251 ± 0.0058; DISCO = 0.8013 ± 0.0078).

**Figure 2:**
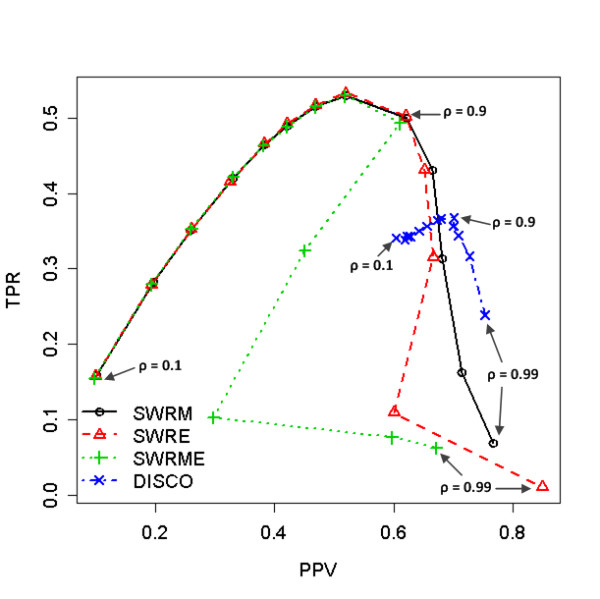
**The effect of cut-off values, ρ, in case of homogeneous data (Spiked-in data)**. The true positive rates (TPR) versus the predictive positive values (PPV) are plotted. The performances of four different methods, SWRM, SWRE, SWRME, and DISCO, are compared according to the thirteen different values, (0.1, 0.2, 0.3, 0.4, 0.5, 0.6, 0.7, 0.8, 0.9, 0.93, 0.95, 0.97, 0.99). The maximum F1 scores are occurred at ρ = 0.9 for all the method.

Figure 2 summarizes the TPR and PPV of the four alignment algorithms using the spiked-in sample data. In this case, the maximum F1 scores are occurred when *ρ *= 0.9 for all the peak alignment methods (as also shown in Figure S2 (b) in the Additional File 1). Likewise, all the three proposed methods show the similar performance to each other in terms of F1 score, while DISCO performs worst as depicted in Figure 2 (F1 score: SWRM = 0.5512 ± 0.0236; SWRE = 0.5526 ± 0.0184; SWRME = 0.5404 ± 0.0320; DISCO = 0.4821 ± 0.0121). The F1 scores of the proposed methods are similar to each other up to the point *ρ *= 0.9. However, when *ρ *is greater than 0.9, SWRM shows the better performance than SWRE and SWRME.

Differently from the previous data, DISCO performs better than the proposed methods when *ρ *goes to 1. In addition, as *ρ *increases, the PPVs of SWRE and SWRME methods decrease when *ρ *is near 0.95. The detailed information of the performance of these two data can be found in the Additional File 2.

In general, SWRM aligns the peak lists of both homogeneous data with higher F1 scores than other methods, and its F1 score is significantly different from that of DICSO. However, it seems that DISCO is less sensitive to the choice of the cut-off value, *ρ*, than the proposed methods since the distributed region of it performances is much narrower than these of the proposed SW-based algorithms as shown in Figures 1 and 2 (also as shown in Figure S2 in the Additional File 1).

### Heterogeneous GC × GC-MS pairwise peak alignment

**Figure 3:**
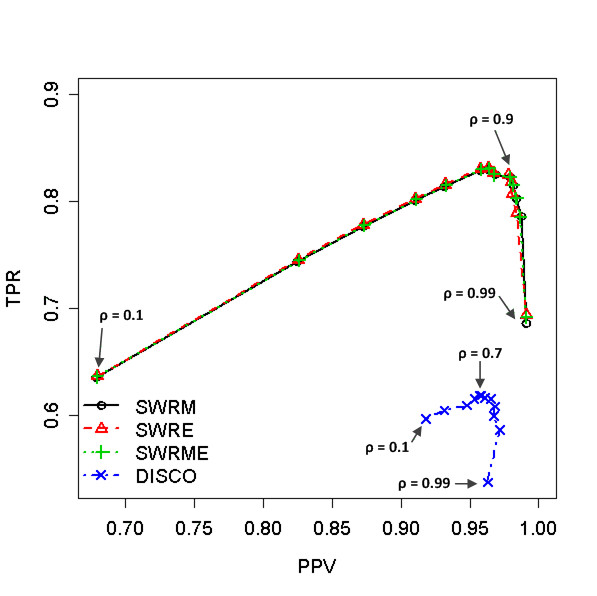
**The effect of cut-off values, ρ, in case of heterogeneous data (Compound standards data)**. The true positive rates (TPR) versus the predictive positive values (PPV) are plotted. The performances of four different methods, SWRM, SWRE, SWRME, and DISCO, are compared according to the thirteen different ρ values, (0.1, 0.2, 0.3, 0.4, 0.5, 0.6, 0.7, 0.8, 0.9, 0.93, 0.95, 0.97, 0.99). The maximum F1 scores are occurred at ρ = 0.9 for SW-based methods and ρ = 0.7 for DISCO.

Figure 3 summarizes the performance of the peak alignments when applied to the heterogeneous data. In order to construct the pairs of the heterogeneous data, we used the three chromatogram sets generated from the different temperature gradients - 5°C/min (run id: S1~S10), 7°C/min (run id: S11~S12), and 10°C/min (run id: S13~S16) - by considering one as a target sample and the other as a reference sample. Similar to the homogeneous data of a mixture of compound standards analyzed at 5°C/min, the proposed methods outperform against DISCO in terms of maximum F1 scores (maximum F1 score: SWRM = 0.8937 ± 0.0032; SWRE = 0.8945 ± 0.0036; SWRME = 0.8937 ± 0.0035; DISCO = 0.7505 ± 0.0057) when *ρ *= 0.9 for SW-based algorithms *ρ *= 0.7 for DISCO and as shown in Figure S2 (c) in the Additional File 1. The F1 scores of DISCO are distributed in a smaller region than those of the proposed methods as the peak alignment with homogeneous data. Overall, the peak alignment of the heterogeneous data is improved by the proposed methods. It should be noted that the proposed methods do not need landmark peaks and retention time transformation for correcting the position of the peaks, while DISCO uses a local linear fitting method along with a z-score based transformation by the landmark peaks.

### The likelihood-based optimal pairwise peak alignment

In order to optimize the peak alignment in terms of F1 score, two likelihood functions, the sum of all the similarity scores of the aligned peak pairs (LS) and the product of all the similarity scores (LP), were designed to reflect the information of F1 score. LS and LP are defined as follows:

where *ρ *is the cut-off value, *q *is the index of the method used: *q *= 1 for SWRM, *q *= 2 for SWRE, and *q *= 3 for SWRME, and *x_i _*and *y_i _*are the *i*th pair of the *k *aligned peak pairs, 1 ≤ *i *≤ *k min *(*m, n*), |*X*| = *m*, |*Y*| = *n *given *ρ *and *q*.

**Figure 4:**
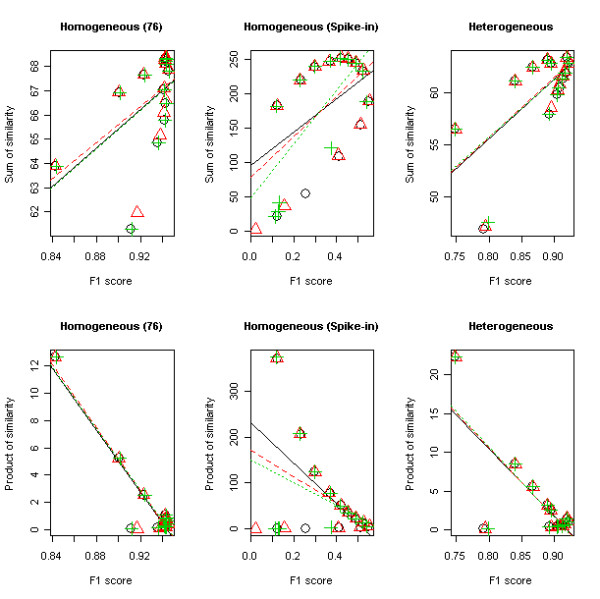
**The scatter plots between F1 score and the likelihood functions**. The upper row is the relationships between the sum of similarity (LS) and F1 score and, in the bottom row, the product of similarity (LP) and F1 score are depicted using compound standards homogeneous data (first column), spiked-in homogeneous data (second column), and compound standards heterogeneous data (last column). In each plot, the black circle and solid line are for SWRM method, the red triangle and dashed line for SWRE method, and the green plus ('+') and dotted line for SWRME method. The lines represent the linear regression fitting.

To study which of these two likelihood functions has the information enough to be an alternative measure of F1 score, we calculated Pearson's correlation coefficients between these two likelihoods and the F1 scores estimated from the homogeneous and heterogeneous data using the proposed three peak alignment methods. Table 2 and Figure 4 summarize these correlations as well as their *p*-values. Theoretically, as the performance of the peak alignment becomes better, LS and LP are increased, suggesting that the correlation with F1 score should be positive for both LS and LP. However, LP was always negatively correlated with F1 score while LS was positively correlated (Table 2 and Figure 4). For this reason, the LS-based likelihood function was used as a surrogate measure of F1 score.

**Table 2:** The correlations between F1 score and the likelihood functions.

	**Homogeneous**				**Heterogeneous**	
		
	**Compound standards**		**Spiked-in**		**Compound standards**	
	**LS**	**LP**	**LS**	**LP**	**LS**	**LP**
			
**SWRM**	0.5501 (0.0515)	***-0.9393 ***(0.0000)	0.4525 (0.1205)	***-0.5951 ***(0.0319)	***0.6849 ***(0.0098)	***-0.7622 ***(0.0025)
**SWRE**	***0.5537 ***(0.0496)	***-0.9561 ***(0.0000)	***0.6015 ***(0.0297)	-0.4515 (0.1214)	***0.6947 ***(0.0084)	***-0.7812 ***(0.0020)
**WRME**	0.5494 (0.0518)	***-0.9389 ***(0.0000)	***0.7034 ***(0.0073)	-0.3612 (0.2252)	***0.6715 ***(0.0120)	***-0.7812 ***(0.0016)

The Pearson's correlation coefficients are calculated between F1 score and the two likelihood functions, the sum of the similarity scores (LS) and the product of the similarity scores (LP), for each method of the proposed peak alignment algorithms, SWRM, SWRE, and SWRME, on two homogeneous data and one heterogeneous data which were generated from a mixture of compound standards and the spiked-in data. The correlations in bold and italic are statistically significant at 5% level (p-value < 0.05) and the values in parentheses are p-value.

An optimal version of the proposed peak alignment methods was then implemented using the LS-based likelihood function. In detail, the optimal version first estimates the optimal choice for the cut-off value and the alignment method from the three proposed methods - SWRM, SWRE, and SWRME - based on the likelihood function, LS:

where *ρ *is the cut-off value; *q *is the index of the method;  and  are the optimal version. Then the pairwise alignment is rendered based on the optimal cut-off value, , and the selected alignment method, .

Based on the algorithm described above, we tested the pairwise peak alignment for two homogeneous and one heterogeneous data. Table 3 shows the maximum and optimal F1 scores for the pairwise peak alignment to compare the performance of the optimal versions. Compared with the proposed SW-based methods, F1 scores of the optimal versions are less but their differences are small. Furthermore, the optimal versions significantly outperform against DISCO as shown in Table 3.

**Table 3:** The maximum and optimal F1 scores of the pairwise peak alignment.

			**SW-based methods**			
		**SWRM**	**SWRE**	**SWRME**	**DISCO**	**Optimal version LS**
			
	**Compound** **standards**	0.9455 (0.0047)	0.9457 (0.0048)	0.9461 (0.0047)	0.8777 (0.0055)	0.9449 (0.0048)
**Homogeneous**	**Spiked-in**	0.5512 (0.0236)	0.5526 (0.0184)	0.5404 (0.0320)	0.4821 (0.0121)	0.4437 (0.0130)
			
**Heterogeneous**	**Compound** **standards**	0.8937 (0.0032)	0.8945 (0.0036)	0.8937 (0.0035)	0.7505 (0.0057)	0.8894 (0.0045)

The maximum F1 scores are estimated for three proposed SW-based methods (SWRM, SWRE, and SWRME) and DISCO, and the optimal F1 scores for the optimal version (LS) of the proposed SW-based methods on two homogeneous and one heterogeneous data. The values in parentheses are standard errors.

## Discussion

Our goal is to develop an improved peak alignment algorithm for both homogeneous and heterogeneous GC × GC-MS data. To achieve this, we adapted the Smith-Waterman local alignment algorithm by modifying its traceback procedure. In addition, we established an optimal version of the SW-based peak alignment algorithms using the sum or product of similarities of aligned peaks as the likelihood function.

Comparing with the only published algorithm DISCO for both homogeneous and heterogeneous peak alignment on the GC × GC-MS data, the proposed algorithms have several differences on aligning the peaks. First, the distance information is not utilized directly in the proposed algorithms, while it plays an important role in DISCO to find the best matched peak pairs. Instead, the proposed approach assumes that the elution order of compounds in the two dimension GC column remains the same across different experiments. This assumption can be a potential issue on the SW-based algorithms since several studies addressed that relative component elution may be affected by temperature and temperature program used [27]. In fact, we observed that when the SW-based methods were applied to the spiked-in sample data, which are much more complicated than the compound standards data, the performance were decreased rapidly. Despite this potential issue, the F1 scores of the SW-based methods even for the spiked-in sample are greater than those of DISCO as shown in Table 3. Nevertheless, some metabolite peaks may not be aligned due to the assumption of constant elution order in the two dimension GC columns. Recently, Mommers and his colleagues [28] introduced the retention time locking (RTL) procedure for the GC × GC-MS experiment, resulting in minimizing the retention time shifts for both dimensions. The SW-based algorithms may be less suffered from the modified elution orders if the comprehensive two-dimensional GC experiments are rendered together with RTL.

Second, DISCO needs to find the landmark peak for estimation of the local linear fitting to correct the retention times. As a result, the quality of the landmark will influence the performance of the peak alignment of DISCO.

Third, no retention time transformation is required in the SW-based peak alignment algorithms. In case of DISCO, once the landmark peaks are selected, the local linear fitting is estimated based on the selected landmark peaks. Therefore, if the retention time shift of the landmark peaks could not accurately reflect the retention time shift of the other metabolites, the local linear fitting will not be able to accurately determining the true retention time shift and result in poor performance of the peak alignment.

The proposed algorithms are free from these difficulties since any transformation and the landmark peaks are not involved. For instance, Figures S3 to S6 in the Additional File 1 display the pairwise alignments for homogeneous and heterogeneous chromatograms of the compound standards data using the SW-based algorithms and DISCO with the pairs of the peak list, *(S1, S10), (S1, S11), (S1, S13)*, and *(S11, S13)*, where *SN *is the run id as described in Table 1. While DISCO aligned the peak pairs after correcting the retention times in Figure S5, the SW-based methods aligned the metabolite peaks without correcting the retention times and employing any transformation. Nevertheless, the proposed SW-based methods clearly performed better than DISCO in terms of F1 scores, demonstrating the advantage of the proposed approaches. The detailed results of these peak alignments can be found in the Additional File 3.

The proposed methods obviously prevail against DISCO for both of homogeneous and heterogeneous data in terms of the maximum F1 score as seen in Figures 1 to 3. DISCO seems to be less sensitive to the cut-off values of the similarity than the SW-based algorithms, however, since its TPR and PPV spanned a narrower range. This is because the role of the cut-off value is different for each method. That is, in DISCO, the cut-off value is used to construct the similarity-based window for the variation in the similarity. Then the peak pair with the smallest distance is chosen as the best matched peak. On the other hand, the SW-based methods take advantage of the cut-off values for building the similarity function *w*(*i, j*) as shown in Equation (2). Namely, as the cut-off value *ρ *decreases, the number of matched peak pairs increases since a peak pair is considered as the peaks originated from the identical compound if their spectral similarity score is greater than *ρ*. That may be the reason that the SW-based methods are much more sensitive to the cut-off value than DISCO since the cut-off value of DISCO is used only to construct the variation window.

In order to ensure the best performance of the alignment, users have to choose an optimal cut-off value for the mass spectral similarity. In reality, it is not easy to find the optimal cut-off value since the optimal value can be data specific. For example, the optimal cut-off value was 0.8 for the compound standards homogeneous data and 0.9 for the spiked-in sample. To overcome this limitation, an optimal version of the SW-based algorithms was established for the automatic peak alignment, where the optimal alignment is established based on the associated likelihood function. In general, the optimal version has the similar performance to the proposed SW-based algorithm and the better performance than DISCO as depicted in Table 3. However, in case of the spiked-in sample, DISCO performed better that the optimal version although the SW-based methods prevailed against DISCO. This may indicate that the likelihood solely with the spectral similarity can recover only partial information of F1 score. Therefore, we may need to incorporate other information such as peak distance into the likelihood for better performance.

## Conclusions

We propose novel peak alignment algorithms capable of aligning both homogeneous and heterogeneous metabolite peaks from GC × GC-MS experiments. Furthermore, we established an automated optimal peak alignment for the proposed algorithms using the likelihood function derived from the sum of the similarities of the aligned peaks. We then demonstrated that the proposed approaches performed better than the existing algorithm DISCO. The main advantage of the proposed approaches is that it can align metabolite peaks for both homogeneous and heterogeneous GC × GC-MS data without the transformation of retention times and the selection of landmark peaks.

## Competing interests

The authors declare that they have no competing interests.

## Authors' contributions

SK developed the algorithms; AF generated the GC × GC-MS data; SK and IK implemented the algorithms; SK and XZ designed and drafted manuscript, approved by all authors.

## Supplementary Material

Additional file 1 **Figures S1, S2, S3, S4, S5, and S6 are in this file**. The density and scatter plots of the two data are depicted in Figure S1. Figure S2 displays F1 scores over the different cut-off value, *ρ*. The homogenous and heterogeneous peak alignments are plotted for four pairs of compound dataset in Figures S3, S4, S5, and S6 for each peak alignment method. Click here for file

Additional file 2 **The results of the pairwise peak alignment for each alignment method are in this file**. The TPR, PPV, and F1 score are reported for four peak alignment algorithms including DISCO according to the different cut-off values applied on the homogeneous and heterogeneous two-dimensional GC data. Click here for file

Additional file 3 **The compound names aligned by the proposed methods and DISCO for Figures S3, S4, S5, and S6**. Click here for file
