# Supplementary material for: Smith-Waterman peak alignment for comprehensive two-dimensional gas chromatography-mass spectrometry
Source: BMC Bioinformatics. 2011 Jun 15;12:235. doi: 10.1186/1471-2105-12-235 (PMC3133553; doi:10.1186/1471-2105-12-235)

Figure S1. The scatter and density plots of the first and the second retention times of the peak list of each dataset. The scatter and density plots for two sets of data are depicted in (a) for compound standards with 5°C/min, (b) 7°C/min, (c) 10°C/min, and (d) for a spiked-in sample. The total number of GCxGC-MS experiments is 10, 2, 4, and 5 for compound standards with 5°C/min, 7°C/min, 10°C/min, and for a spiked-in sample, respectively.

(a)

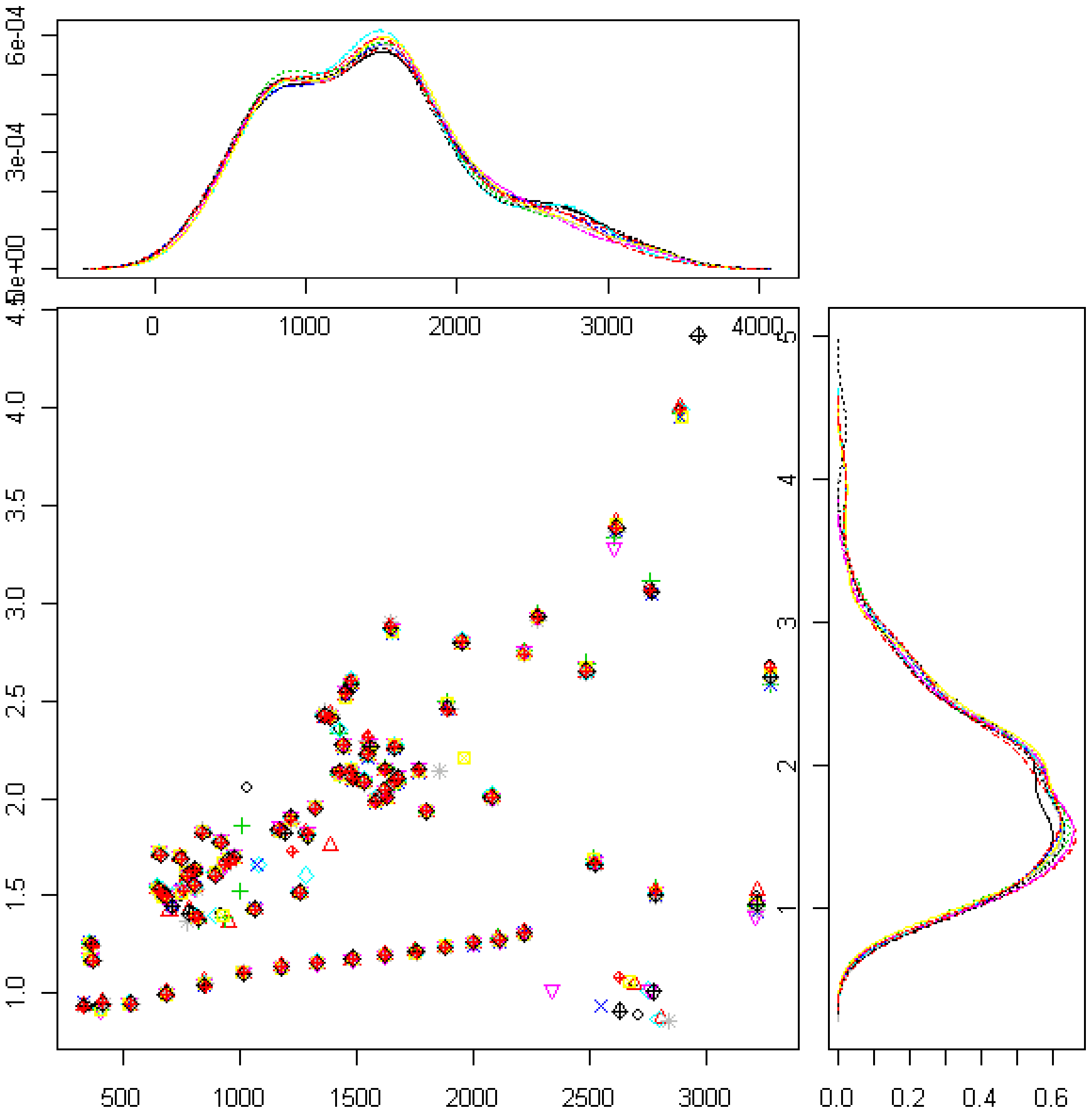

(b)

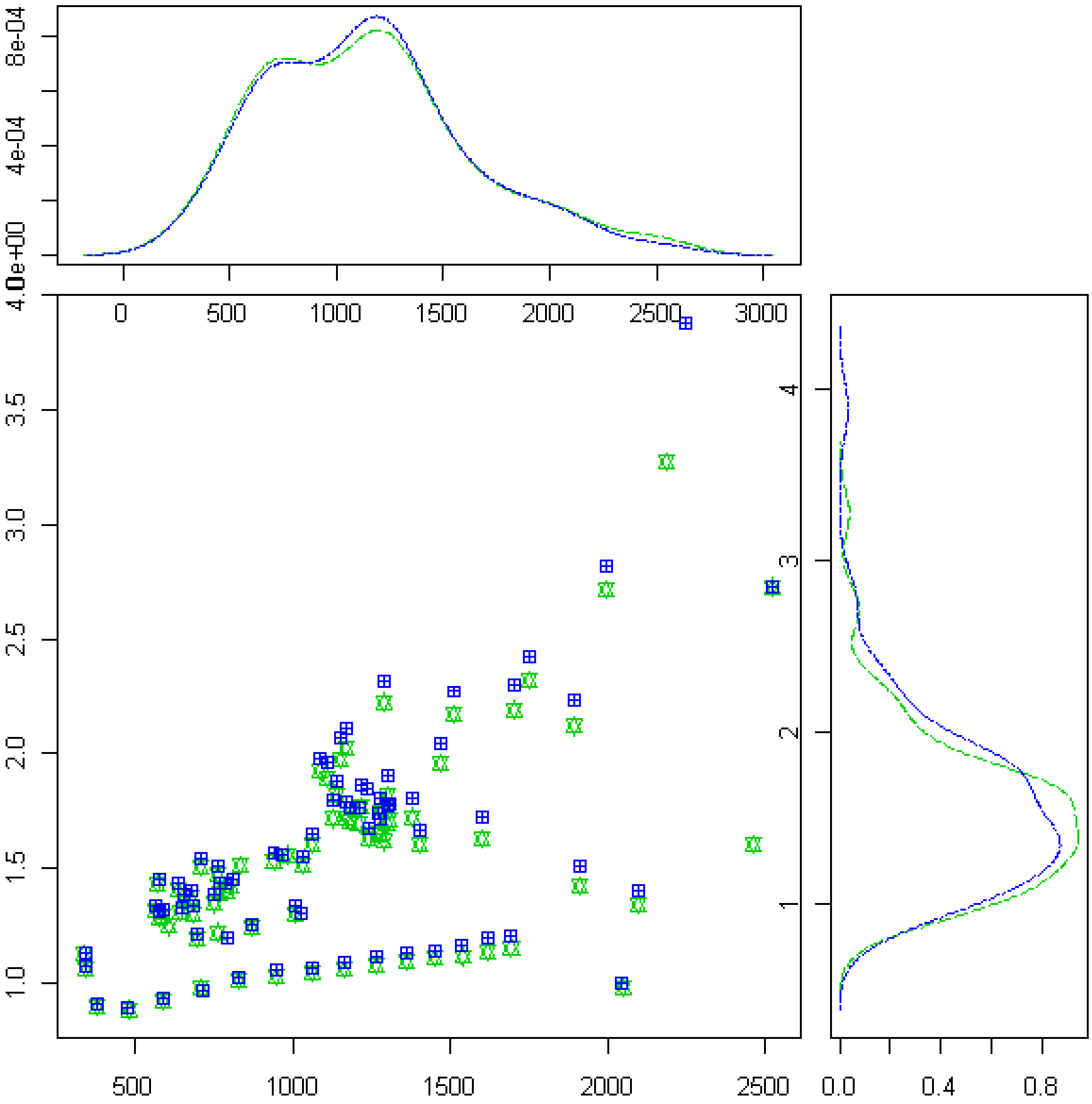

8e-04
4e-04
0.0e+00
0
500
1000
1500
2000
2500
3000
4.0
3.5
3.0
2.5
2.0
1.5
1.0
500
1000
1500
2000
2500
4
3
2
1
0.0
0.4
0.8

(c)

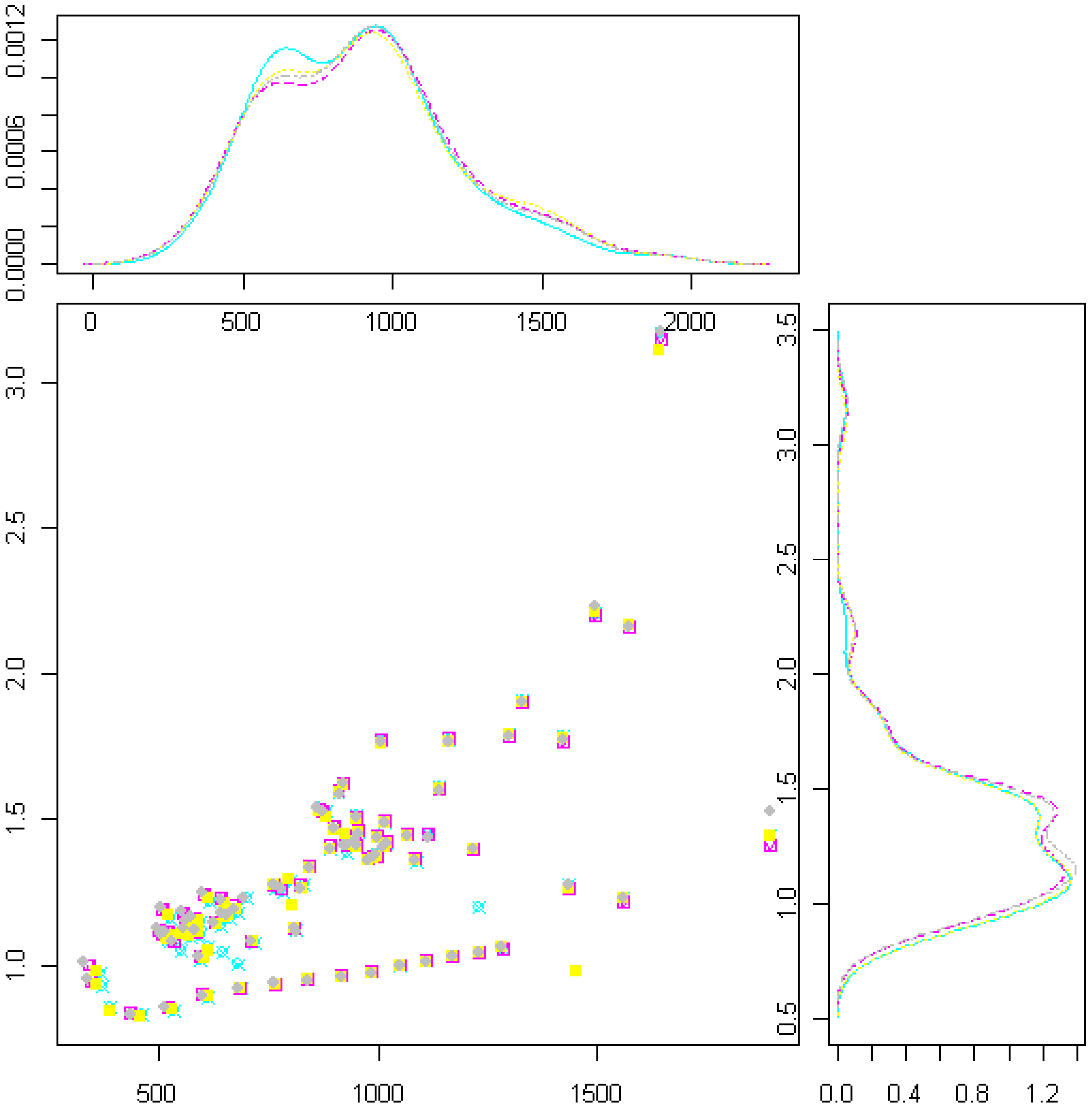

0.0000
0.0006
0.0012
0
500
1000
1500
2000
1.0
1.5
2.0
2.5
3.0
3.5
0.5
0.0
0.4
0.8
1.2

(d)

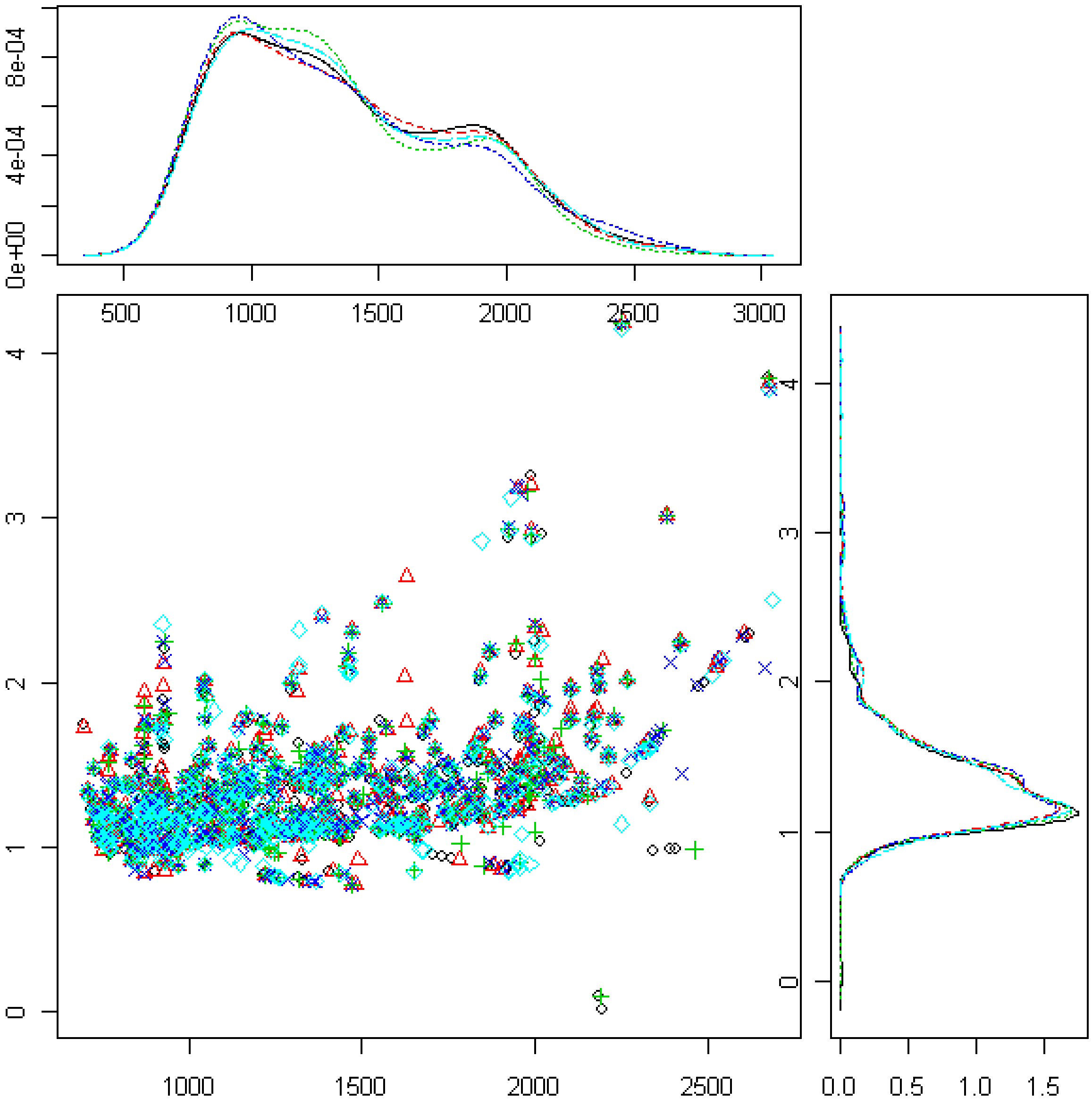

8e-04
4e-04
0e+00
500
1000
1500
2000
2500
3000
4
3
2
1
0
1000
1500
2000
2500
0.0
0.5
1.0
1.5

Figure S2. The F1 scores of pairwise alignments for homogeneous and heterogeneous chromatograms. (a) The homogeneous peak alignment of compound standards. (b) The homogeneous peak alignment of a spiked-in sample. (c) The heterogeneous peak alignment of compound standards.

(a)

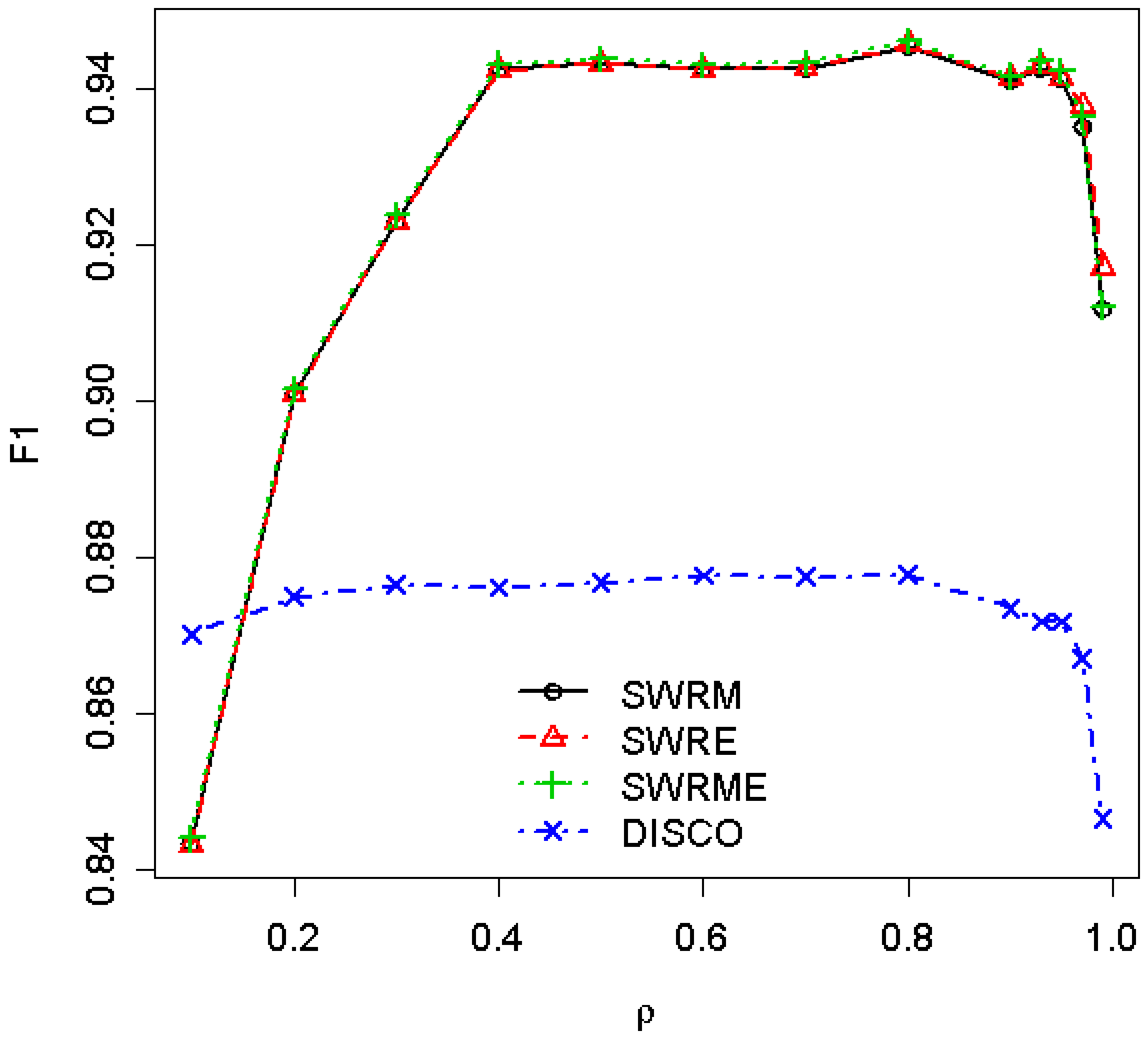

(b)

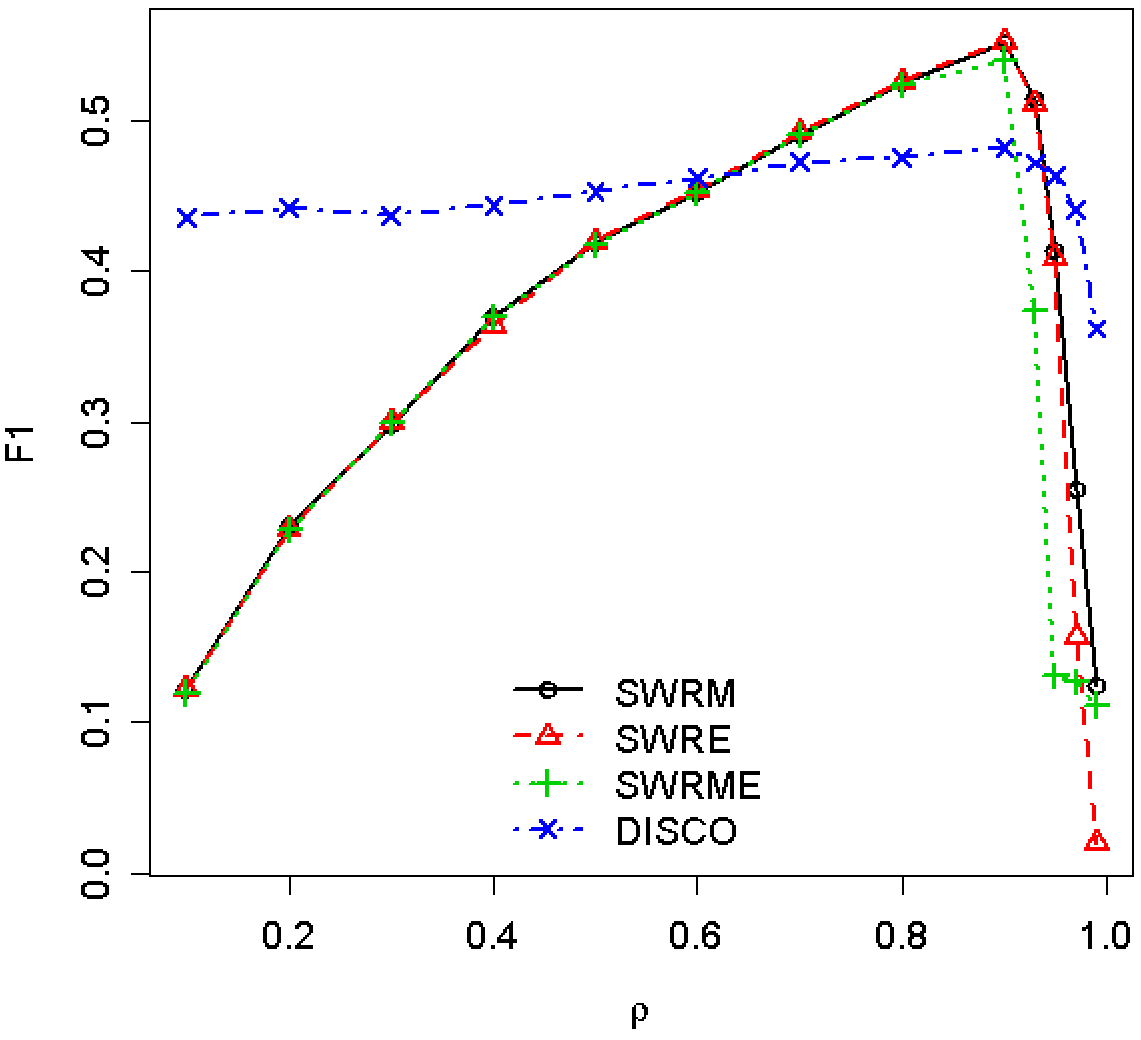

(c)

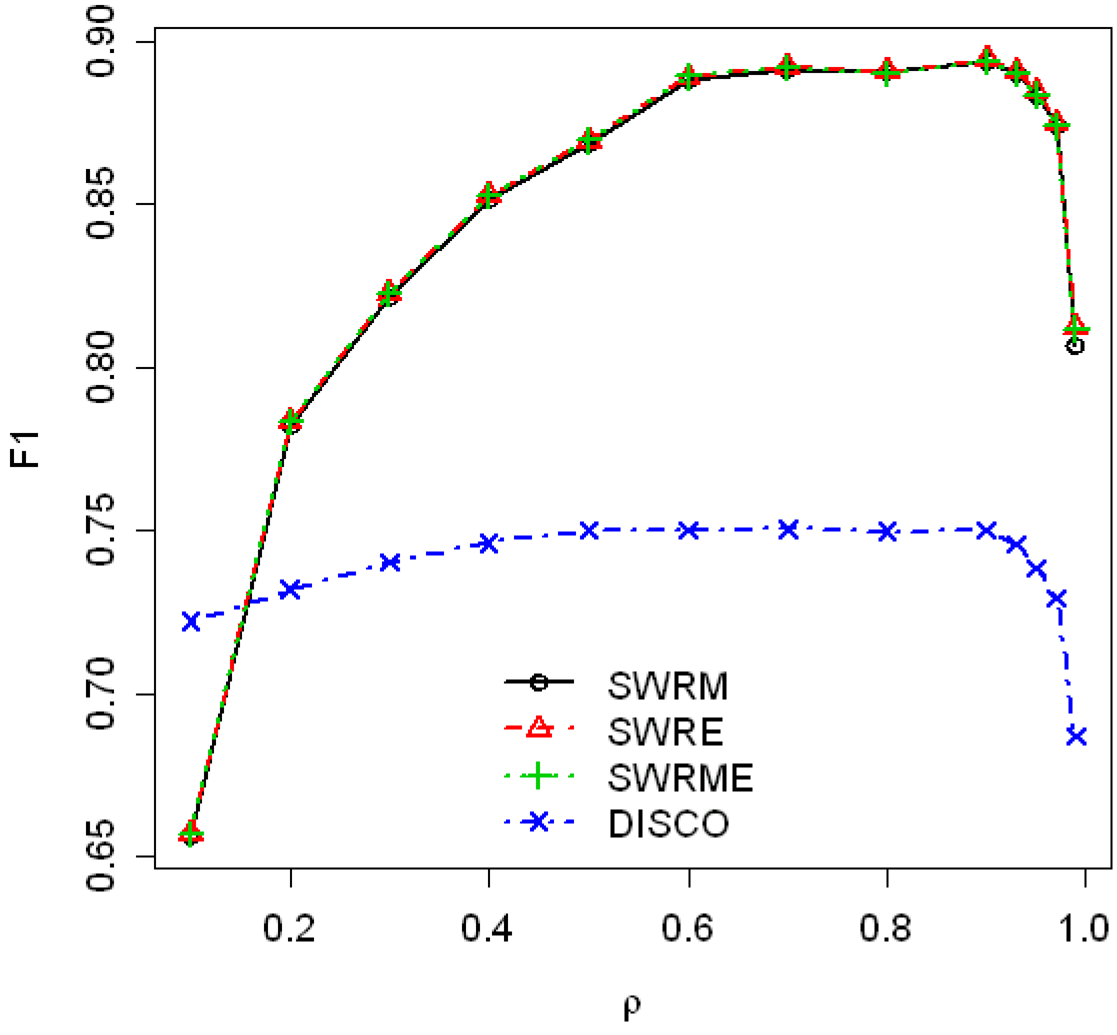

Figure S3. The pairwise alignments for homogeneous and heterogeneous chromatograms of compound standards data using SWRM. (a) The homogeneous peak alignment between S1 (5 °C/min) and S10 (5 °C/min). (b) The heterogeneous peak alignment between S1 (5 °C/min) and S11 (7 °C/min). (c) The heterogeneous peak alignment between S1 (5 °C/min) and S13 (10 °C/min). (d) The heterogeneous peak alignment between S11 (7 °C/min) and S13 (10 °C/min).

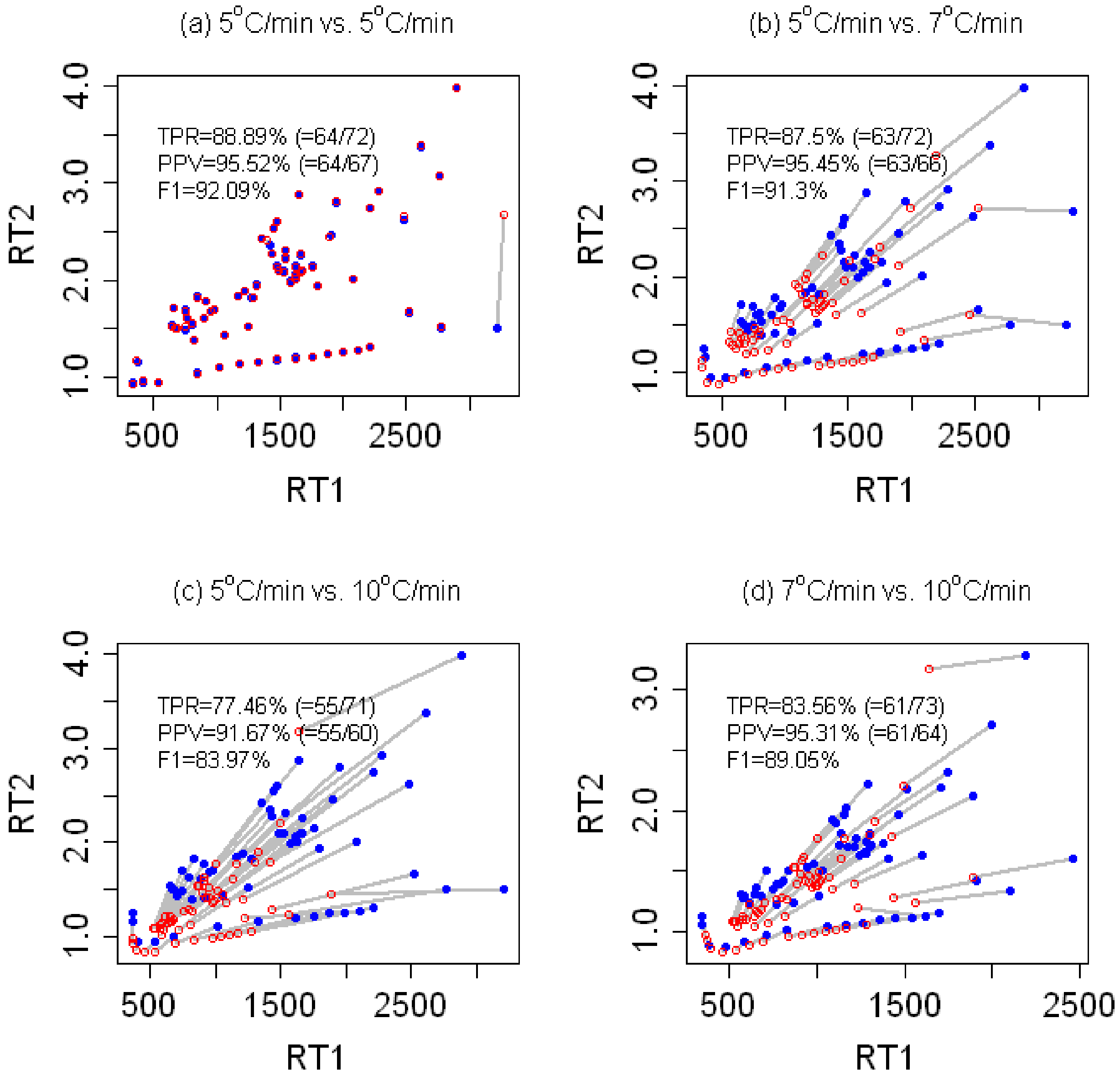

Figure S4. The pairwise alignments for homogeneous and heterogeneous chromatograms of compound standards data using SWRE. (a) The homogeneous peak alignment between S1 (5 °C/min) and S10 (5 °C/min). (b) The heterogeneous peak alignment between S1 (5 °C/min) and S11 (7 °C/min). (c) The heterogeneous peak alignment between S1 (5 °C/min) and S13 (10 °C/min). (d) The heterogeneous peak alignment between S11 (7 °C/min) and S13 (10 °C/min).

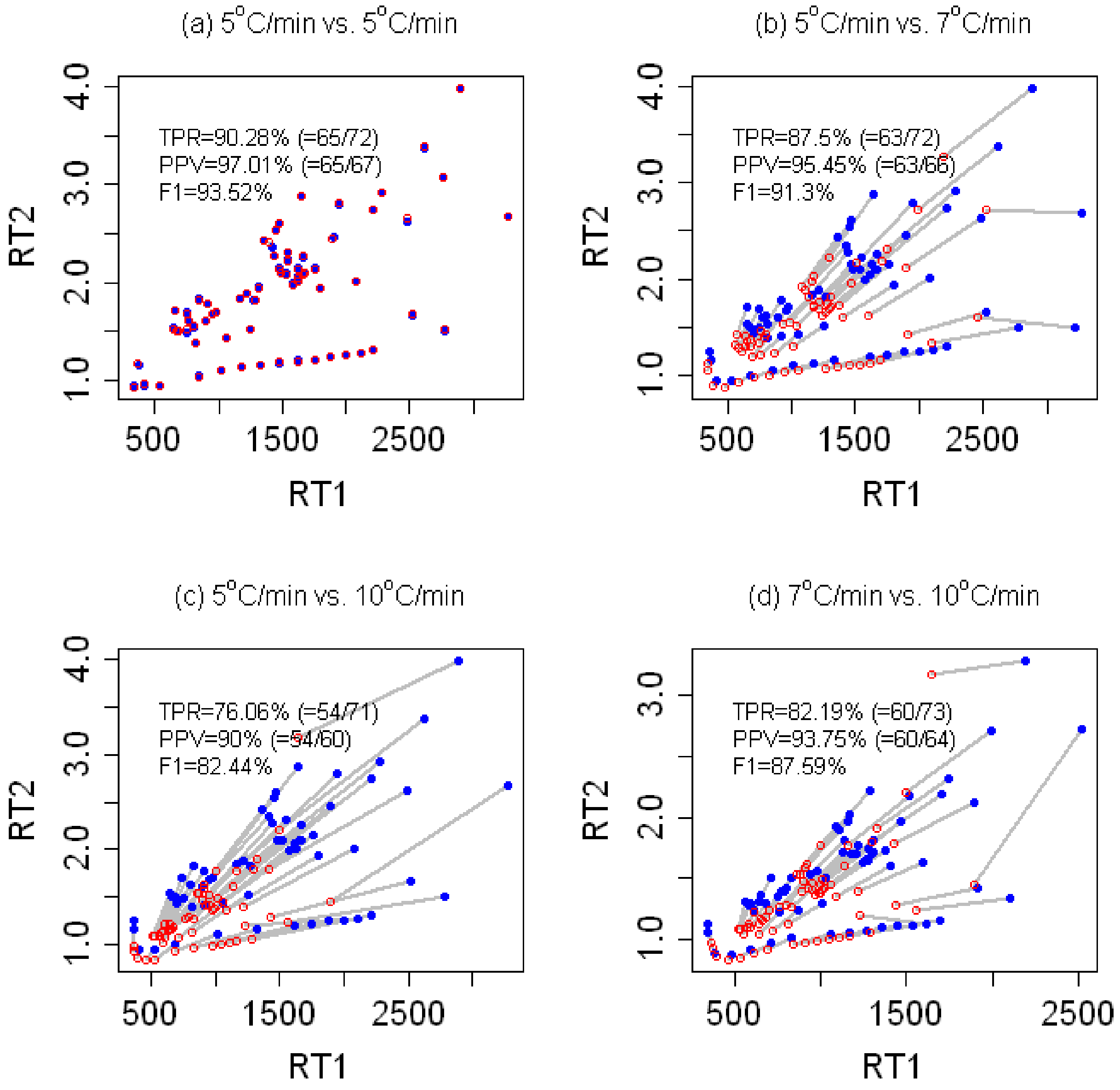

Figure S5. The pairwise alignments for homogeneous and heterogeneous chromatograms of compound standards data using SWRME. (a) The homogeneous peak alignment between S1 (5 °C/min) and S10 (5 °C/min). (b) The heterogeneous peak alignment between S1 (5 °C/min) and S11 (7 °C/min). (c) The heterogeneous peak alignment between S1 (5 °C/min) and S13 (10 °C/min). (d) The heterogeneous peak alignment between S11 (7 °C/min) and S13 (10 °C/min).

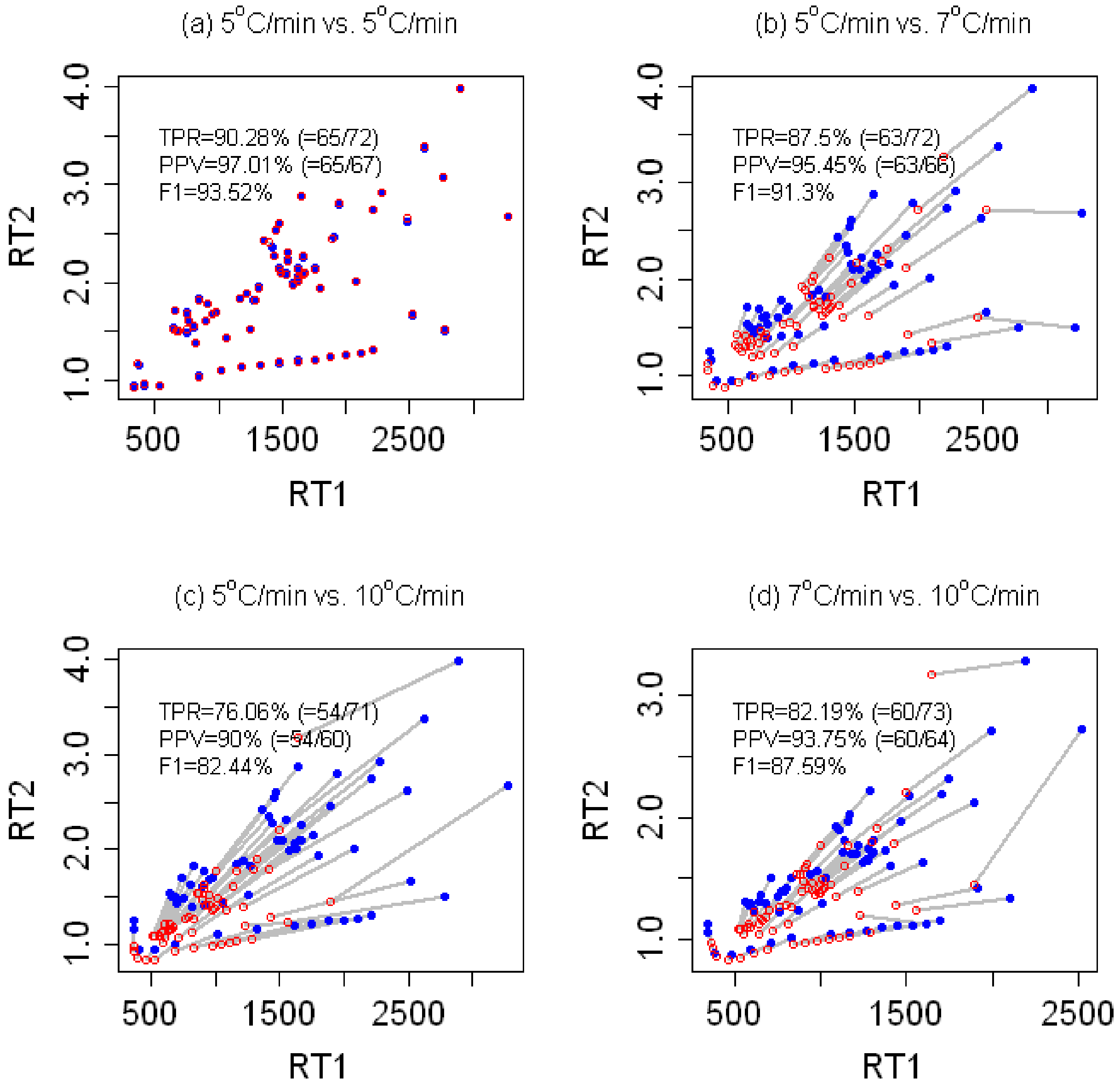

Figure S6. The pairwise alignments for homogeneous and heterogeneous chromatograms of compound standards data using DISCO. (a) The homogeneous peak alignment between S1 (5 °C/min) and S10 (5 °C/min). (b) The heterogeneous peak alignment between S1 (5 °C/min) and S11 (7 °C/min). (c) The heterogeneous peak alignment between S1 (5 °C/min) and S13 (10 °C/min). (d) The heterogeneous peak alignment between S11 (7 °C/min) and S13 (10 °C/min).

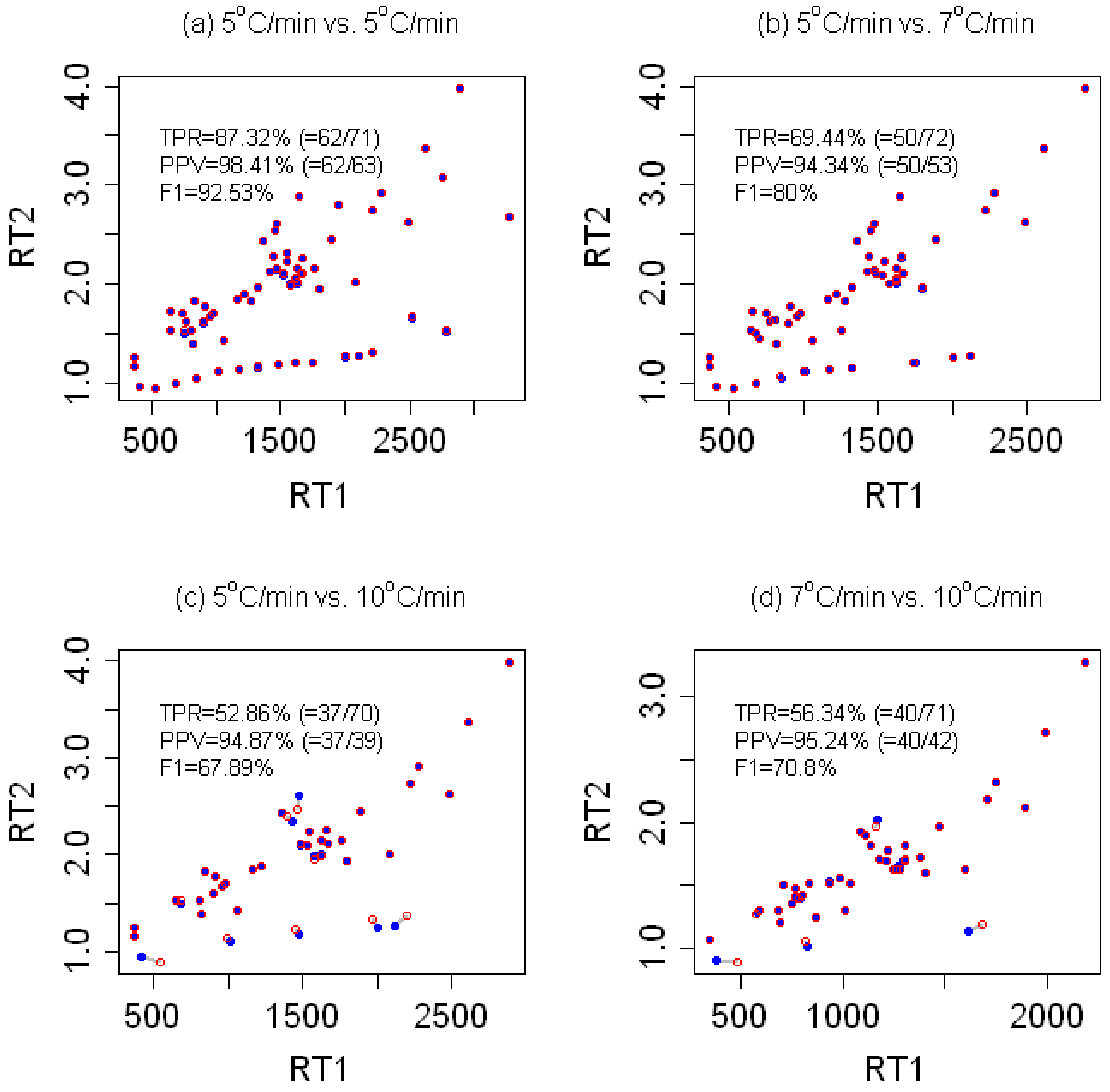

Supplement: Additional file 1 — Figures S1, S2, S3, S4, S5, and S6 are in this file. The density and scatter plots of the two data are depicted in Figure S1. Figure S2 displays F1 scores over the different cut-off value, ρ. The homogenous and heterogeneous peak alignments are plotted for four pairs of compound dataset in Figures S3, S4, S5, and S6 for each peak alignment method. [file 1471-2105-12-235-S1.PDF]
